# Efficient Generation of Myostatin Knock-Out Sheep Using CRISPR/Cas9 Technology and Microinjection into Zygotes

**DOI:** 10.1371/journal.pone.0136690

**Published:** 2015-08-25

**Authors:** M. Crispo, A. P. Mulet, L. Tesson, N. Barrera, F. Cuadro, P. C. dos Santos-Neto, T. H. Nguyen, A. Crénéguy, L. Brusselle, I. Anegón, A. Menchaca

**Affiliations:** 1 Unidad de Animales Transgénicos y de Experimentación (UATE), Institut Pasteur de Montevideo, Montevideo, Uruguay; 2 Instituto de Reproducción Animal Uruguay, Fundación IRAUy, Montevideo, Uruguay; 3 INSERM UMR 1064, Center for Research in Transplantation and Immunology-ITUN, Nantes, France; Institut Jacques Monod, FRANCE

## Abstract

While CRISPR/Cas9 technology has proven to be a valuable system to generate gene-targeted modified animals in several species, this tool has been scarcely reported in farm animals. Myostatin is encoded by *MSTN* gene involved in the inhibition of muscle differentiation and growth. We determined the efficiency of the CRISPR/Cas9 system to edit *MSTN* in sheep and generate knock-out (KO) animals with the aim to promote muscle development and body growth. We generated CRISPR/Cas9 mRNAs specific for ovine *MSTN* and microinjected them into the cytoplasm of ovine zygotes. When embryo development of CRISPR/Cas9 microinjected zygotes (n = 216) was compared with buffer injected embryos (n = 183) and non microinjected embryos (n = 173), cleavage rate was lower for both microinjected groups (P<0.05) and neither was affected by CRISPR/Cas9 content in the injected medium. Embryo development to blastocyst was not affected by microinjection and was similar among the experimental groups. From 20 embryos analyzed by Sanger sequencing, ten were mutant (heterozygous or mosaic; 50% efficiency). To obtain live *MSTN* KO lambs, 53 blastocysts produced after zygote CRISPR/Cas9 microinjection were transferred to 29 recipient females resulting in 65.5% (19/29) of pregnant ewes and 41.5% (22/53) of newborns. From 22 born lambs analyzed by T7EI and Sanger sequencing, ten showed indel mutations at *MSTN* gene. Eight showed mutations in both alleles and five of them were homozygous for indels generating out-of frame mutations that resulted in premature stop codons. Western blot analysis of homozygous KO founders confirmed the absence of myostatin, showing heavier body weight than wild type counterparts. In conclusion, our results demonstrate that CRISPR/Cas9 system was a very efficient tool to generate gene KO sheep. This technology is quick and easy to perform and less expensive than previous techniques, and can be applied to obtain genetically modified animal models of interest for biomedicine and livestock.

## Introduction

Modification of the genome in large animals has always been a difficult task, with few efficient technologies available until recently [1–4]. The generation of genetically modified large animals has several advantages for different applications not only in meat, dairy or wool production but also in the generation of bioreactors to produce recombinant proteins of biomedical interest or for the production of models of human diseases [5]. Nevertheless, the massive application of these technologies has been limited due in part to the low efficiency to obtain transgenic founders [6, 7]. While lentiviral transgenesis does show high efficiency [8], site-specific gene integration is still not possible, among other problems. Gene edition in general and in particular the generation of genetically-modified animals has experienced a major advance in the last few years due to the incorporation of novel technologies of direct microinjection into zygotes of gene-specific nucleases such as zinc finger nucleases (ZFN) [9], TALENs [10] and homing endonucleases [11, 12]. Although the use of these gene-specific nucleases made possible the generation of KO animals, they are difficult to engineer. Lately, a new gene-specific nuclease is being applied with high efficiency. Known as clustered regularly interspaced short palindromic repeats (CRISPR/Cas9), this system is taken from the adaptive immune system of bacteria and archae [13], and is based in small RNAs and Cas endonucleases proteins that can induce site-specific DNA double-stranded breaks. CRISPR/Cas9 has been rapidly tested in several species to date [14–19] and promises to revolutionize the field of genome editing in general and in particular transgenic vegetal and animal fields. Genetically-modified farm animals using zygote microinjection of CRISPR/Cas9 have been reported recently in pigs [20], goats [17] and sheep [21], but more information is needed to extend the generated information.

According to official data of FAO estimations, in order to feed a larger, more urban and richer human population, food production must be increased around 70 percent before the year 2050 [22]. According to meat production, the improvement of traditional animal breeds with modern technologies should be implemented. Myostatin is a member of the transforming growth factor beta (TGF-β) superfamily, involved in the inhibition of muscle differentiation and growth [23]. Previous studies have reported that the inhibition of this protein induces a significant increase in the muscle volume and mass producing more meat in animals known as double-muscle animals [24–27]. In farm animals, sheep are good models to be modified genetically due to small size and short gestation period compared to cows. However, until now their use was limited due to the lack of efficient gene editing tools in livestock. In addition, the silencing of this gene in breeds as Australian Merino, the most specialized breed in the production of superfine or ultrafine wool, could be an interesting model to produce more meat in a high-quality wool producing animal.

The objective of this study was to demonstrate the efficiency of the CRISPR/Cas9 gene editing technology coupled to zygote microinjection in the generation of *MSTN* KO sheep. We show that this approach induces mutations with a high efficiency (45.5%), resulting in live offspring carrying single-gene mono and bi-allelic mutations.

## Materials and Methods

### Ethics statement

All procedures that include animal handling were carried out in strict accordance with the recommendations in the Guide for the Care and Use of Laboratory Animals of the National Institutes of Health. Protocol # 001/2014 was approved by the Animal Care Committee of the Fundación IRAUy certified by the National Council of Animal Care of Uruguay and have therefore been performed in accordance with the ethical standards laid down in the 1964 Declaration of Helsinki and its later amendments. All surgeries were performed under strict aseptic conditions, and all efforts were made to minimize animal suffering.

### Generation of the plasmid co-expressing Cas9 and sgRNA

The pX330-U6-Chimeric_BB-CBh-hSpCas9 plasmid (a gift from Feng Zhang, Addgene plasmid # 42230) was digested with BbsI, dephosphoryated using Antarctic Phosphatase (NEB, UK), and the linearized vector was gel purified. To generate the bicistronic vector (pX330-cas9-MSTN) expressing Cas9 and sgRNA against *MSTN* (GGCTGTGTAATGCATGCTTG), a pair of oligos for targeting myostatin exon 1 (5’-CACCGGCTGTGTAATGCATGCTTG-3’ and 5’-CACCGGCGAAGCTTACTGAGGATT-3’) was annealed, phosphorylated and ligated to a linearized vector (details of the vector plasmid is described by Cong et al [28]).

### Genome editing assay in cells

The A15 astroglial sheep cell line (kindly provided by J. Chapuis, UR 892, INRA, Jouy-en-Josas, France) [29] was maintained in Dulbecco’s modified Eagle medium (DMEM) in 10% Fetal Bovine Serum, 2mM glutamine, 1% sodium pyruvate and 1% penicillin/streptomycin. Cells were transfected in 24-well plates with 2μg of pX330-cas9-MSTN co-expressing Cas9 and sgRNA against myostatin using lipofectamine LTX reagent (Life technologies, NY, USA) according to the manufacturer’s manual. Three days later, genomic DNA from transfected cells was extracted using a kit from Macherey Nagel (NucleoSpin Tissue, Duren, Germany) and quantified using the NanoDrop2000 spectrophotometer (Thermo Fisher Scientific, Illkirch, France). A260/A280 and A260/A230 ratios accounted for sample purity.

Gene mutation activity of sgRNA sequence at the target locus of *MSTN* exon 1 was quantified using the T7EI mismatch detection assay. DNA sequence of interest was PCR-amplified with a high-fidelity polymerase (Herculase II fusion polymerase) using specific primers (forward primer 5’-tcactggtgtggcaagttgt-3’ and reverse primer 5’-aaaagctctttgccctcctc-3’). The 634bp PCR product was then denatured and slowly re-annealed (95°C, 2min; 95°C to 85°C, -2°C/sec; 85°C to 25°C, -1°C/sec) to produce homoduplex/heteroduplex mix. This was then digested by 5U of T7EI restriction enzyme (NEB) at 37°C for 30 minutes. Digestion products were separated by 2% agarose gel electrophoresis. The ratio of cleaved (255-bp and 379-bp) to uncleaved (634-bp) products was used to calculate NHEJ frequency as previously described using Image J software [30]. NHEJ frequency was calculated as follow: % gene modification = 100 X (1-(1-fraction cleaved)^1/2.

### Production of sgRNA and Cas9 mRNA

These procedures have been described in detail elsewhere [31]. Briefly, T7 promoter was added to sgRNA template by PCR amplification of pX330-cas9-*MSTN* plasmid using the following primers: 5’-ttaatacgactcactataggctgtgtaatgcatgcttg-3’ and 5’-tttaaaagcaccgactcggtgcc-3’. The PCR product was purified using NucleoSpin Gel and PCR Clean-up (Macherey Nagel). It was used as the template for *in vitro* transcription using MEGAshortscript T7 kit (Life Technologies) according to the manufacturer’s manual. Following completion of transcription, DNase I treatment was performed.

The Cas9 mRNA was transcribed using PmeI-digested Cas9 expression JDS246 plasmid (a gift from Keith Joung, Addgene plasmid # 43861) and the mMESSAGE mMACHINE T7 ULTRA Transcription Kit (Life Technologies) according to the manufacturer’s manual. Following completion of transcription, the poly(A) tailing reaction and DNase I treatment were performed.

Both the Cas9 mRNA and the sgRNAs were purified using MEGAclear kit (Life Technologies) and eluted in elution buffer.

### Prediction of potential Off Target sites

Potential targets of the CRISPR sgRNA *MSTN* were found using the rules outlined in [32] since this is the only web tool that includes the sheep genome for predicting potential off-targets. The sequences TAATGCATGCTTGTGG, TAATGCATGCTTGAGG, TAATGCATGCTTGCGG, TAATGCATGCTTGGGG (underlined is the PAM, preceded by the N13 sequence of the sgRNA MSTN) were blasted in the genome database of the sheep (http://blast.ncbi.nlm.nih.gov/Blast.cgi). Seven potential off-target sites were detected, that were amplified in the DNA samples from all the mutant lambs, and subjected to Sanger sequencing.

### *In vitro* production of embryos

Unless otherwise indicated, chemicals were purchased from Sigma Chemical Company (St Louis, MO, USA). The embryos were produced by *in vitro* fertilization according to routine procedure performed in our lab as described earlier [8]. Briefly, ovaries from slaughterhouse were transported to the laboratory and cumulus oocyte complexes (COCs) were aspirated in recovery medium. The selected COCs were placed in maturation medium for 24 h in 5% CO_2_ in humidified air atmosphere at 39°C. Then, expanded COCs were inseminated in 100 μl drops with 1 x 10^6^ dose of frozen-thawed semen selected by ascendant migration on a swim up method. Fertilization was carried out in 5% CO_2_ with humidified atmosphere at 39°C for 22 h.

### Microinjection into zygotes

Soon after fertilization, 572 presumptive zygotes were randomly assigned to three experimental groups to be microinjected (CRISPR group, n = 216; and Buffer group, n = 183) or not (Control group, n = 173). Microinjection of CRISPR group was performed into the cytoplasm with 5 ng/μl of sgRNA and 20ng/μl of Cas9 mRNA diluted in injection buffer (10mM Tris pH 7.5, 0.1mM EDTA), while Buffer group was injected with the same procedure but with buffer alone. Lastly, injected and non-injected embryos were transferred to culture medium under mineral oil, in 5% CO_2_, 5% O_2_ and 90% N_2_ in humidified atmosphere at 39°C. Cleavage rate on Day 2 (cleaved zygotes per total oocytes) and development rate on Day 6 (morulae and blastocysts per total oocytes) were recorded for all experimental groups. After Day 6, DNA from 20 CRISPR group embryos was analyzed by Sanger sequencing to detect the mutation at the *MSTN* gene level.

To determine the *in vivo* efficiency of the system, 53 blastocysts produced by CRISPR/Cas9 zygote microinjection were transferred to 29 recipient females. Only early blastocysts, blastocysts and expanded blastocysts classified as excellent or good (i.e. Grade 1) [33] were transferred on Day 6 after fertilization. Embryo transfer was performed by minimally invasive surgery assisted by laparoscopy to place the embryos into the cranial side of the ipsilateral uterine horn to the corpus luteum. Recipient ewes were previously synchronized to be on Day 6 of the estrous cycle using a standard protocol to control ovulation described previously [34].

### Monitoring of fetuses and lambs

Pregnancy diagnosis and fetal development were performed on Day 30 and 105, respectively, by using B-mode ultrasonography equipped with a 5 and 3.5 MHz probe (Well-D, Shenzhen, China). Day 0 of the experiment was defined as the moment of embryo fertilization. Several parameters were measured to study the development of fetuses at Day 105 of gestation: thoracic diameter, biparietal diameter, occipitonasal length and heart rate. At delivery, length of gestation, gender, rectal temperature, heart and respiratory rates, body weight, thoracic perimeter, biparietal diameter, crown-rump and occipitonasal length, height at withers, height at hips, width at hips and width at chest were recorded. Body weight and morphometric variables were determined at birth, and 15, 30 and 60 days later.

### Identification and genotyping of transgenic animals

Samples from skin and limb muscle of the lambs were taken seven days after birth and T7EI assay, western blot test and histology examinations were performed in order to identify and characterize KO founders and off-target sites. Total DNA was isolated from skin biopsies for all animals and from muscle for some animals. PCR and T7EI assay were performed using the same protocol than from cells samples. Samples were analyzed using capillary electrophoresis (Caliper, PerkinElmer, Hopkinton, MA). Genotyping of *MSTN* exon 1 was performed by direct sequencing of PCR amplicons using the same primers described above and in muscle biopsies by additional sequencing of isolated bacterial clones with individual amplicon sequences, as described in detail previously [30, 35].

### Analysis of myostatin expression

Western blotting was performed to determine the presence of myostatin in the muscle fiber. Equal amounts of total proteins were run on 12% (v/v) gel electrophoresis and electrophoretically transferred to a PVDF membrane. Monoclonal mouse anti-myostatin (sc-398333, Santacruz) and anti-GAPDH (G8795, Sigma) antibodies were used in the western blotting. The washed membranes were incubated with 1:50000 dilution of secondary antibody linked to horseradish peroxidase (HPR). HPR activity was detected using western blot chemiluminescence (Promega, MA, USA).

### Muscle fiber histology

Samples from deltoid and biceps femoris muscles were fixed in 4% formaldehyde solution until analysis. Samples were included in paraffin, sectioned in 1 mm slides and stained with haematoxilin-eosin to study muscle morphology. Muscle fibers (min 250 per sample) were measured with respect to their minimum Feret (MinFeret) diameter. The mean area was also calculated.

### Statistical analysis

*In vitro* embryo development on Day 2 and Day 6 among experimental groups was compared by logistic regression. Continues variables measured during pregnancy and body growth after birth in mutant *vs*. wild type lambs were analyzed by one-way ANOVA or by non-parametric Kruskal Wallis test. Differences were considered significant when P<0.05.

## Results

### Efficiency of Cas9/sgRNA activity in sheep fetal fibroblast

**Fig 1:**
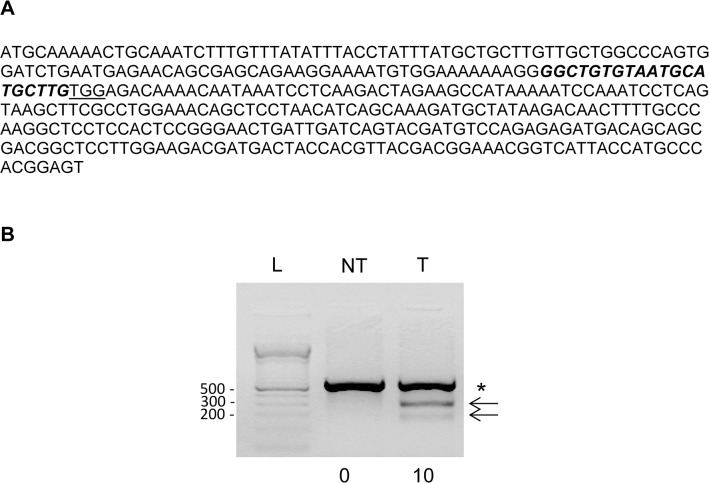
CRISPR-Cas9 genome editing activity in transfected ovine cells. **A)** Exon 1 of the ovine *MSTN* gene. The sequence recognized by sgRNA is in italic and bold, from nt 110–130, the PAM sequence TGG is underlined and the rest of exonic sequences in smaller font. **B)** Ovine cells were transfected with pX330-cas9-*MSTN* plasmid co-expressing Cas9 and sgRNA and three days later cellular DNA was isolated and subjected to T7EI assay for detecting mutation at the targeted *MSTN* exon 1. The PCR amplifies a band of 634 bp and uncleaved (asterisk) and cleaved (arrows, 255 bp and 379 bp) products are indicated. Numbers below each lane indicate the percentage of targeted indels. NT: non-transduced cells, T: transfected cells, L: 1Kb DNA. The experiment is representative of two replicates performed with similar results.

Before microinjection of the sgRNA and Cas9 coding sequences into the ovine zygotes, fetal ovine fibroblasts were transfected with the pX330-cas9-*MSTN* plasmid co-expressing Cas9 and sgRNA, showing mutations at the targeted *MSTN* exon 1 in 10% of the total DNA (Fig 1). Although it is difficult to compare with other cell lines, this efficacy *in vitro* is comparable to what we and others have previously observed as being sufficient to obtain mutated animals by microinjection into zygotes of not only CRISPRs/Cas9 but also ZFNs or TALENs [15, 35]. Thus, we proceeded to microinject ovine zygotes with both mRNAs encoding for Cas9 and sgRNA.

### Analysis of *MSTN* mutations in embryos microinjected with Cas9/sgRNA mRNAs

We first determined whether zygotes microinjected with Cas9 and sgRNA mRNAs and further cultured *in vitro* showed normal development and *MSTN* mutations. From 216 injected zygotes 63.9% survived to micromanipulation and reached cleavage stage on Day 2, and 39.1% achieved morula or blastocyst stage on Day 6. Cleavage rate and development rate for control non-injected embryos were significantly higher than for CRISPR and buffer microinjected embryos (Table 1). However, no differences were found on embryo development rate among control and microinjected zygote groups after cleavage. The total number of embryos on Day 6 from cleaved embryos did not show statistical differences among groups (Table 1). Thus, microinjection resulted in some embryo lethality up to the cleavage state but Cas9 and sgRNA mRNAs microinjection had no adverse effects on subsequent *in vitro* embryo development.

**Table 1:** Cleavage rate on Day 2 and development rate on Day 6 of embryos that were microinjected into cytoplasm with CRISPR/Cas9 RNA system, buffer injection solution, or non-injected embryos (Control group).

	No. zigotes	Cleavage rate on Day 2	Morulae and Blastocysts on Day 6	No. of embryos on Day 6/clived	Mutant embryos at blastocyst stage
CRISPR injection	216	63,9% (138/216)^a^	25,0% (54/216)^a^	39,1% (54/138)^a^	50.0% (10/20)
Buffer injection	183	60,7% (111/183)^a^	20,2% (37/183)^a^	33,3% (37/111)^a^	—
Non-injected	173	86,1% (149/173)^b^	35,8% (62/173)^b^	41,6% (62/149)^a^	—

For different superscripts, P<0.05.

DNA from developed CRISPR injected embryos were extracted and analyzed by Sanger sequencing. From 20 embryos analyzed, 10 showed mutations at the *MSTN* gene (50%). Two of those mutants were heterozygous (wt + mutated allele) and eight were mosaic presenting more than two sequences. The deletions observed ranged from 6 to 350 bp, besides just 1 bp insertions were observed.

### PCR and T7EI genotyping of lambs following microinjection of Cas9/sgRNA mRNAs

**Fig 2:**
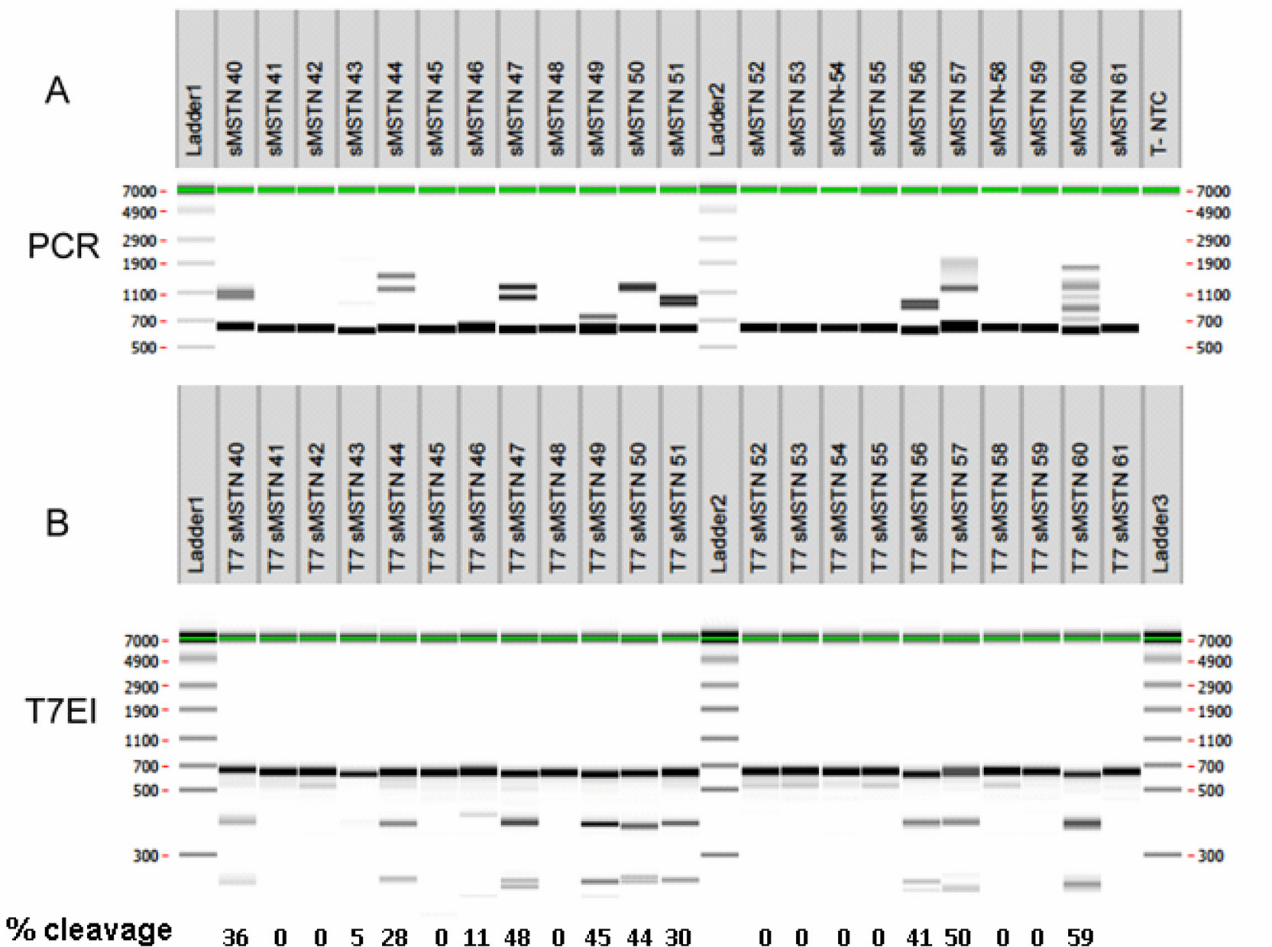
Genotype of lambs born following microinjection of sgRNA for *MSTN* exon 1 and Cas9. DNA was extracted from lambs’ skin biopsies one week after birth, with the exception of lambs #43 and #49 that were stillborn and were biopsied 1–2 hours after delivery. **A)** Results from PCR amplifying 634 bp. Additional larger bands represent formation of heteroduplex DNA double strands with mismatches between the two strands resulting in slower migration during electrophoresis. **B)** T7EI assay of lambs produced by zygote injection of CRISPR/Cas9 mRNA, indicating numbers of mutant animals with bands of 255, 379 and 634 bp (#40, 43, 44, 47, 49, 50, 51, 56, 57 and 60) and WT animals with only one band of 634 bp (#41, 42, 45, 46, 48, 52, 53, 54, 55, 58, 59 and 61).

From 53 Grade 1 *in vitro* produced sheep blastocyst transferred into 29 recipient females, 41.5% of them were detected at 30 days of gestation, 65.5% of the recipients were pregnant and 22 lambs were delivered (Table 2). From these 22 lambs, three (#43 49 and 53) died at delivery or within the first day after birth. Skin and muscle biopsies from deltoid and biceps femoris were taken from 22 lambs within 1 week after birth. DNA from the 22 skin biopsies was extracted and analyzed by PCR and T7EI assay followed by capillary electrophoresis (Fig 2). PCR analysis showed the presence of main band of the expected size in the absence of mutations (634 bp) and bands of apparent higher molecular weight, due to open angles formed by heteroduplexes of DNA strands with mismatches [36] in nine animals, suggesting the presence of mutations (Fig 2A). In an additional animal (#43) a single band of slightly smaller size as compared to the other animals was observed (Fig 2A).

**Table 2:** Efficiency obtained with CRISPR/Cas9 system injected into cytoplasm of ovine zygotes to produce *MSTN* mutant lambs. Data obtained from 53 embryos transferred into 29 recipient ewes.

	Embryos on Day 30	Pregnant ewes	Fetal loss	Mutant/ born lambs	Biallelic/ mutant lambs	Homozygous/ mutant lambs
CRISPR/Cas9 efficiency	41.5% (22/53)	65.5% (19/29)	0.0% (0/22)	45.5% (10/22)	80.0% (8/10)	50.0% (5/10)

Since T7EI digestion of amplicon allows detection of mutations when both DNA strands show mismatches, animals with WT sequences or with the same mutation in both alleles should not generate new bands and animals which have different alleles, either one WT and the other mutated or both with different mutations, should generate new bands. The T7EI assay revealed that the same nine animals with heteroduplexes in the PCR assay generated bands of lower molecular weight upon T7EI digestion (Fig 2B), confirming the presence of mutations in these animals. Animal #43 did not show new smaller bands but if both alleles contained the same mutation the T7EI assay was not expected to be positive. The analysis of DNA cleavage showed that several animals (#47, 49, 50, 56, 57 and 60) had around 50% cleavage corresponding to half of the alleles being mutated and are thus not mosaic. Some animals (#40, 44, 46 and 51) had cleavage of less than 50% and are likely mosaic.

### DNA sequence genotyping of lambs following microinjection of Cas9/sgRNA mRNAs

**Fig 3:**
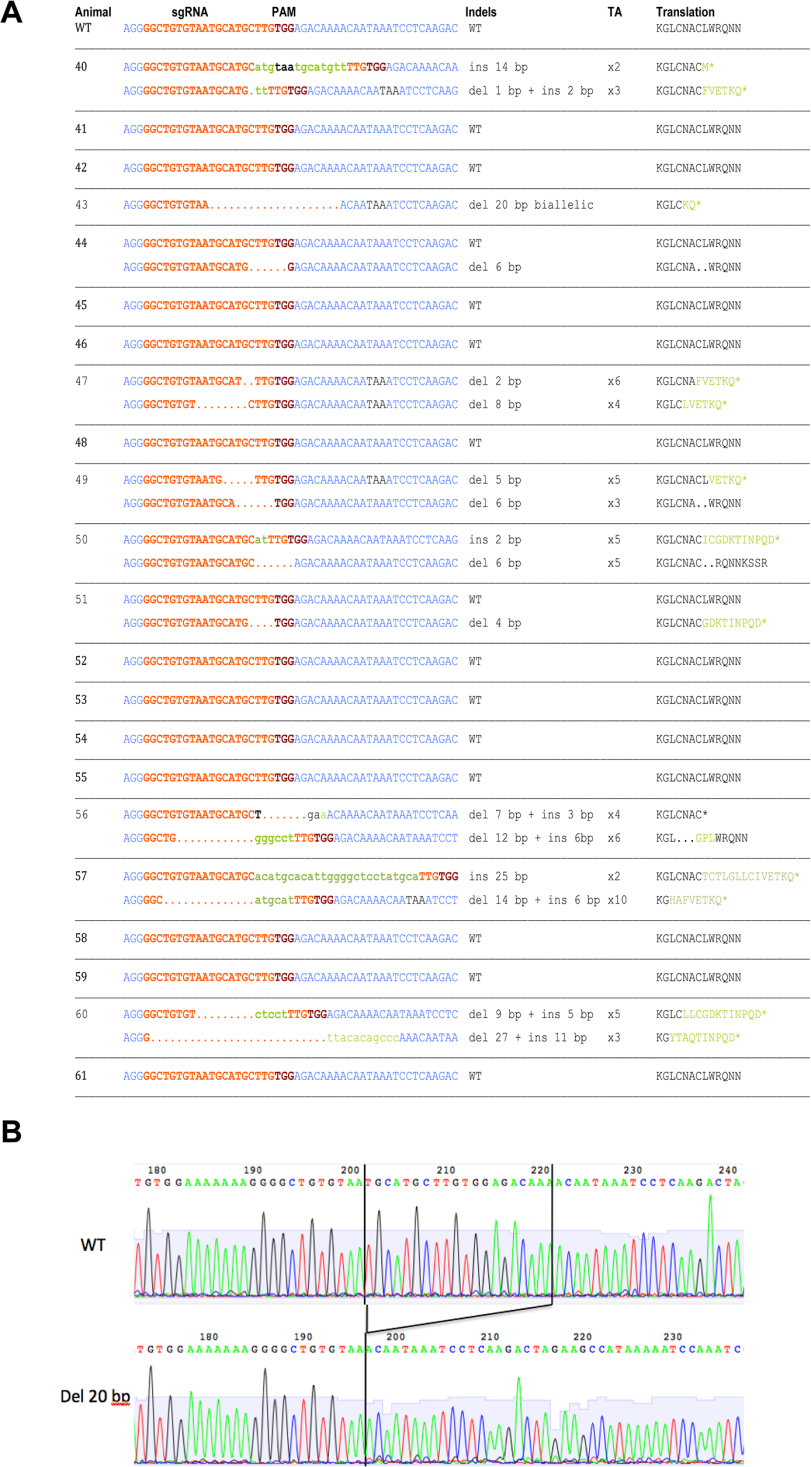
Sequence analysis of lambs’ *MSTN* exon 1. The same DNAs analyzed in Fig 2 were PCR amplified and the amplicons directly sequenced. In some animals (#40, 47, 49, 50, 56, 57 and 60) DNA from muscle biopsies were PCR amplified and amplicons were cloned into plasmids by TA cloning and electroporated into bacteria, followed by sequencing of 8–10 bacterial clones. **A)** Depicts for each of the 22 delivered lambs the flanking DNA sequences (in blue) close to the targeted sgRNA sequence (in red) and the PAM sequence (violet); missing nucleotides are represented by spaces, added ones in green and small characters and stop codons are labeled in black. The column Genotype recapitulates the genotype found for each lamb. The column TA indicates the number of bacterial colonies that were sequenced for each allele of the muscle biopsies. The column Translation depicts the aminoacids translated; spaces for the missing ones, in green the ones that are new due to the shift in the coding reading frame and the * represents the stop of the aminoacid sequence due to the premature stop codons. Results are representative of two different PCR amplicons sequencing for all animals. **B)** A representative sequence electrophoresis, in this case the one of animal #43 which has a biallelic identical deletion of 20 nt.

Sequencing of PCR amplicons compassing the targeted *MSTN* exon 1 sequence showed that ten animals contained mutations; nine identified using capillary electrophoresis and animal #43 which showed an identical deletion of 20 nt in both chromosomes (Fig 3A and 3B), explaining the smaller size of the amplicons in capillary electrophoresis and the negative T7EI assay. The other 12 animals showed only WT alleles. Among the ten mutated animals, eight showed both alleles mutated (#40, 43, 47, 49, 50, 56, 57 and 60) and two only one allele (#44 and 51). These introduced mutations disrupted the coding frame of exon 1 (number of indels different of 3nt or multiples of 3nt) with the generation of premature stop codons in nine mutated animals in at least one of the two alleles. The exception was animal #44 which showed an in-frame shift mutation (Fig 3A). Newly generated stop codons (Fig 3A) are recognized by cells as premature stop codons due to the absence of other normal *Cis* sequences targeting these mRNAs for degradation through nonsense-mediate decay pathway resulting in the complete absence of the protein [36]. Some animals showed biallelic frameshift mutations introducing premature stop codons (#40, 43, 47, 57 and 60), and are thus homozygous KO animals. Other animals showed one frameshift mutation and another with inframe mutations (#49, 50 and 56) and are thus heterozygous KO animals. Analogously, one animal (#51) showed one allele with a frameshift mutation and a WT allele and thus it is also a heterozygous KO animal. Finally, one animal (#44) had one allele with an inframe mutation and one wild-type allele and thus it is not a KO animal.

The DNA sequences performed in skin and muscle from the same lambs showed the same mutations (S1 Table), indicating the absence of mosaicism in ectoderm and mesoderm-derived tissues and thus indicating the action of Cas9 at the zygote stage. Thus, sgRNA and Cas9 mRNA microinjection into ovine zygotes achieved high efficient introduction of mutations resulting in inactivation of the *MSTN* gene.

Primers pairs were designed and the possible off target sites were amplified and sequenced in order to assess the presence of possible off target effects (S2 Table). In the animals #40 and #57 one 27 pb deletion and one 1 bp insertion were found respectively for the off target region number 3. In both cases this mutation was heterozygous. None other off target effects were identified in the seven regions assessed.

### Analysis of myostatin expression

**Fig 4:**
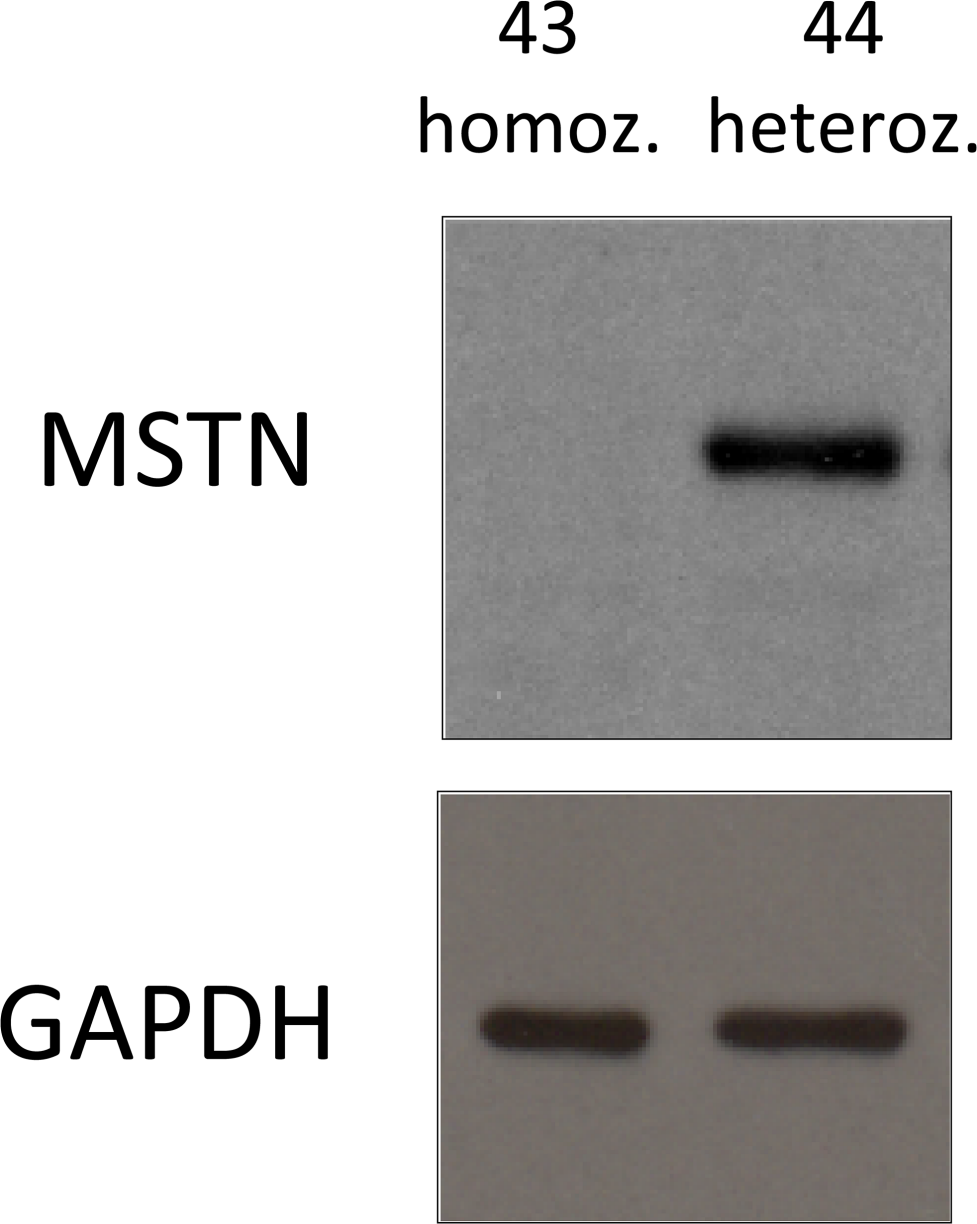
Myostatin western blot. Muscle biopsies from two representative animals, one homozygous (homoz.) for a frameshift mutation and another heterozygous (heteroz.) for a frameshift mutation and a WT copy in the second allele (# 43 and 44, respectively, the numbers of animals correspond to those of Fig 3) analyzed by western blot using an anti-MSTN monoclonal antibody. After stripping of the anti-MSTN antibody from the membrane, an anti-GAPDH was used as loading control. One representative western blot experiment out of three performed with the same results.

Homozygous KO lambs with two frameshift mutations should show absence of myostatin whereas heterozygous KO lambs with one coding allele should show myostatin. Western blot for myostatin analysis showed undetectable levels in homozygous KO lamb #43 and the presence of myostatin in heterozygous (1 WT and 1 in-frame shift allele) lamb #44 (Fig 4) and these levels were comparable to those of lambs with 2 WT alleles (S1 Fig).

### Fetal development and delivery

Fetal determinations by ultrasonography on Day 105 of gestation showed no statistical differences for thoracic and biparietal diameter, occipito-nasal length or heart rate among WT, homozygous KO or heterozygous KO mutant animals (S2 Fig), indicating that the mutation did not affect *in utero* measurements although the *MSTN* gene was already mutated.

For mutant lambs, # 43 and 49 died at delivery and lamb # 57 died within first month after birth due to a wound infection. For WT lambs, #53 and 58 died at delivery and within first month after birth, respectively. The rest of the lambs developed normaly until at least 60 days of life. Length of gestation was 147.0 ± 0.9, 149.8 ± 0.6 and 149.4 ± 1.0 days for WT, homozygous KO and heterozygous KO mutant lambs respectively (P = NS). Rectal temperature at birth was 40.2 ± 0.2, 39.9 ± 0.3 and 39.9 0078 0.4°C for WT, homozygous KO and heterozygous KO mutant lambs, respectively (P = NS). Heart rate and respiratory rate did not differ among groups and was 189.8 ± 3.1 and 93.3 ± 1.8 for WT lambs, 184.0 ± 5.7 and 86.0 ± 13.1 for homozygous KO lambs, and 203.0 ± 11.8 and 75.0 ± 5.0 for heterozygous KO lambs, respectively.

### Postnatal phenotype

**Fig 5:**
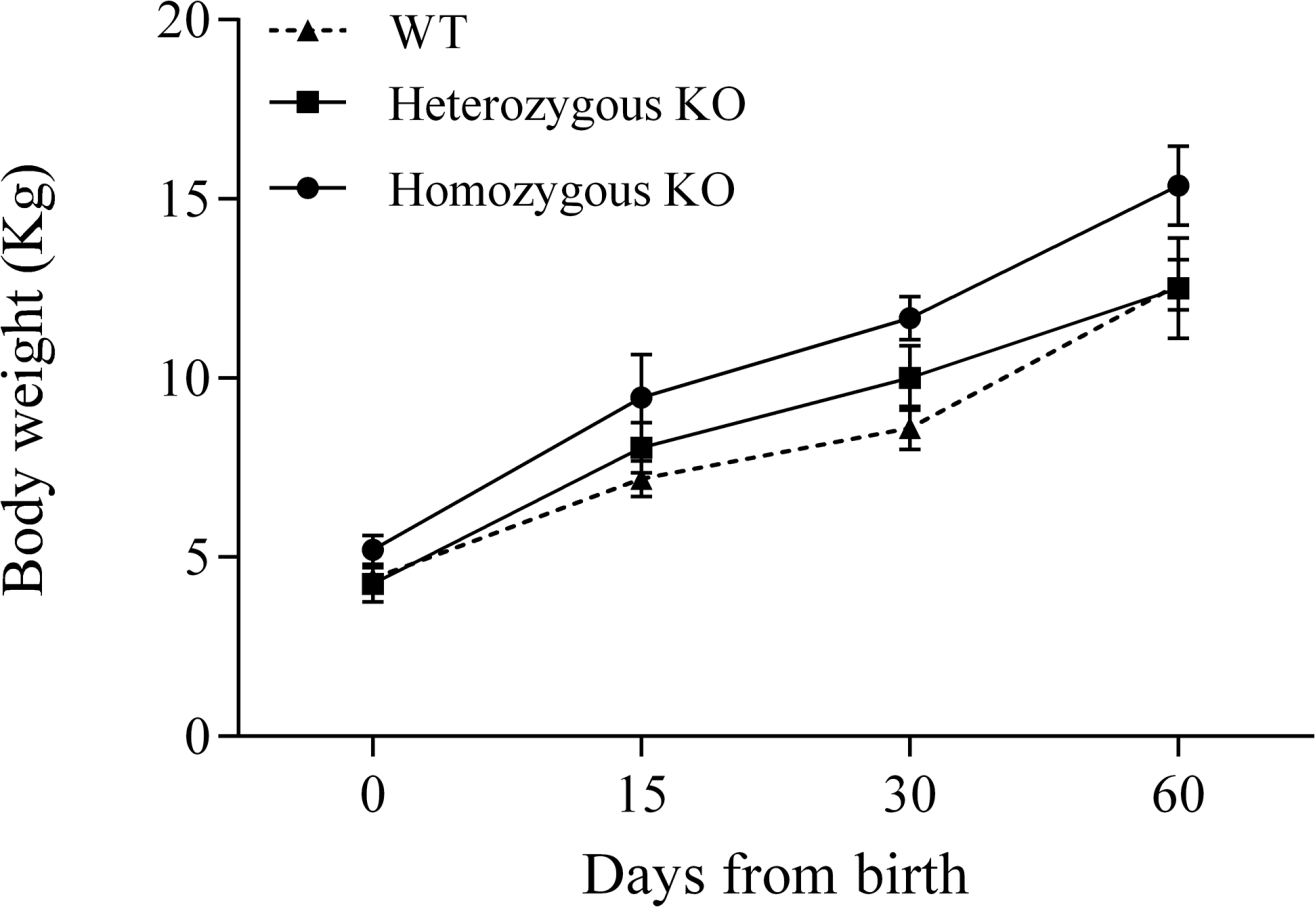
Body weight in lambs produced by CRISPR/Cas9 system. Significant diference (P<0.05) from 15 to 60 days after birth was found among homozygous KO and wild-type lambs.

**Fig 6:**
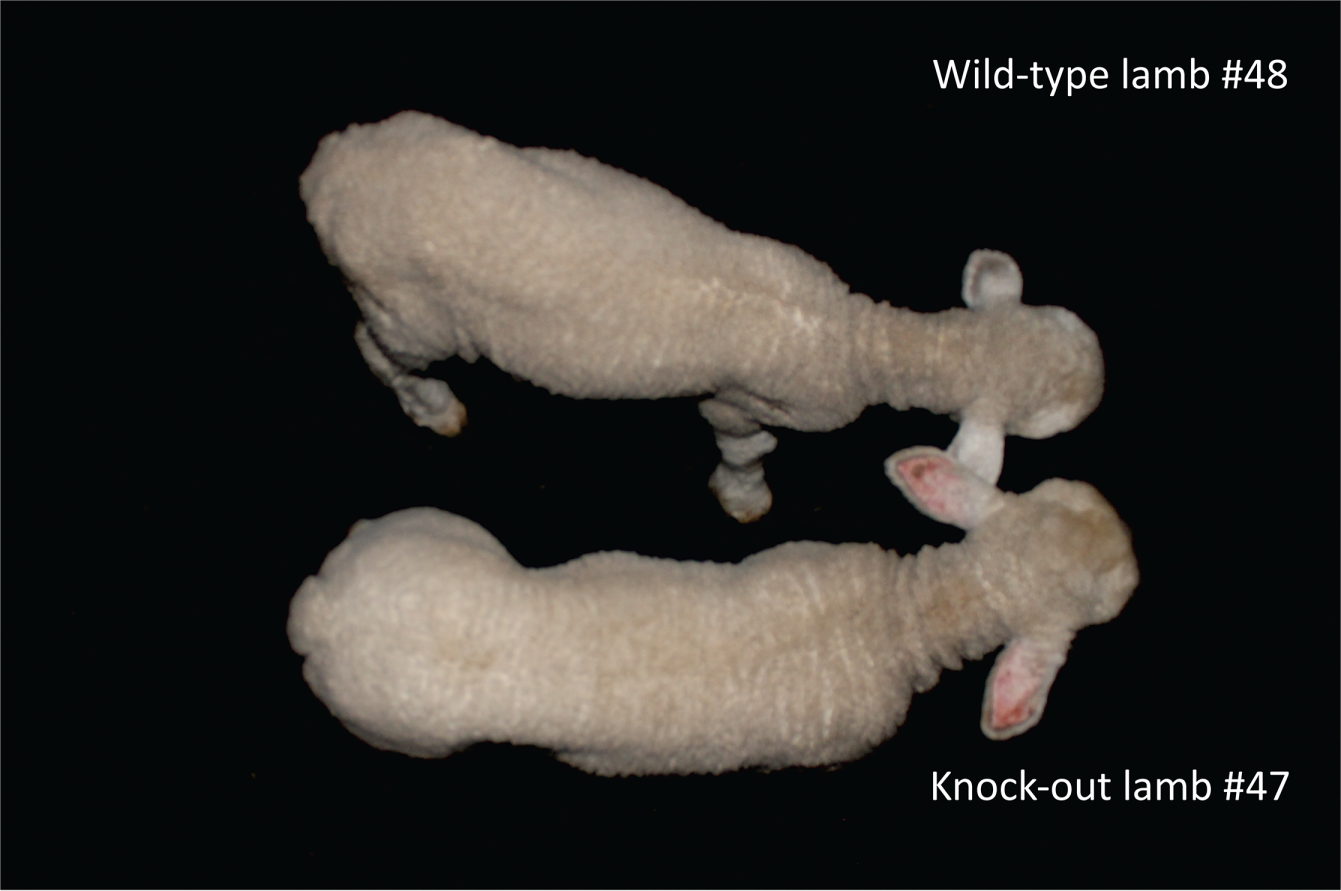
Phenotype of lambs 30 days after birth. The lamb at the top is wild type (#48), the lamb at the bottom is a homozygous KO (#47). Note the differences in muscle mass from the posterior limbs and loin from the homozygous KO lamb when compared to wild-type. At the time of this picture, with 30 days old the body weight was 8.750 (#48) and 11.150 kg (#47).

Morphometric variables registered from birth to 60 days later are shown in Fig 5 and S3 Fig. Body weight from homozygous KO lambs was not different than WT animals at birth (P = NS) and was greater at 15 and 30 days (P<0.05) with a statistical tendency at 60 days after birth (P = 0.08). This faster growth showed by homozygous KO lambs within two months of age averaged an increase between 20 and 30%. Body weight for heterozygous KO mutant lambs was intermediate (P = NS). The rest of the variables did not show statistical differences among the three genotypes, indicating that the mutation at the *MSTN* gene affects body weight mainly by increasing muscle mass (S3 Fig). However, a statistical tendency (P<0.1) was found at 60 days for height and width at withers, and width at chest among homozygous KO and WT lambs, which gave a very clear phenotype in some *MSTN* KO lambs as shown in Fig 6.

### Muscle fiber histology

As expected from previous results of lambs with spontaneous *MSTN* mutations [37], histological analyses of muscle biopsies showed muscle fibers of lower diameter although due to the relatively low number of homozygous KO animals analyzed (n = 4) it did not reach statistical significance when compared to WT animals (data not shown).

## Discussion

The current study demonstrates that microinjection into zygotes of sgRNA and Cas9 mRNAs was a very efficient approach to obtain myostatin KO sheep. Embryo survival and embryo development were not affected by the microinjection of sgRNA and Cas9 mRNAs into the cytoplasm. Also, no differences in fetal loss, birth rate and postnatal survival rates were detected for both groups, suggesting that mutant animals are as healthy as WT animals. These results are in agreement with our recent report where the microinjection of a GFP lentiviral construct into ovine zygotes did not affect embryo development, fetal growth or postnatal variables when compared to WT lambs [8]. Ten lambs with mutations in the *MSTN* gene were obtained (45.5%), eight had biallelic mutations and five of them were biallelic out of frame shift mutations. These lambs resulted in myostatin deficiency and a double-muscle phenotype with a significant increase in body weight when compared to control wild type animals.

During recent years gene editing nucleases have revolutionized the field of transgenesis, decreasing the time, cost and effort to obtain genetically-modified animals [38]. However, the use of CRISPR/Cas9 system has not been reported in many ruminant species to date [17, 38, 21]. We demonstrated that this technology does not affect embryo survival and *in vitro* development rate when injected into the cytoplasm of zygotes. However, the cleavage rate was affected by the embryo manipulation required for the microinjection *per se*, which has been reported previously in zygotes submitted to zone pellucidae break and microinjection [39]. No effect of microinjection was found in the ability of those surviving embryos 48h after fertilization to continue its development and to reach blastocyst stage on Day 6 after fertilization. In addition, the overall procedure allowed a high fertility rate since 41.5% of the microinjected embryos transferred to recipients were pregnant and developed to term. The efficiency of the technique is also seen in the fact that no gestation loss occurs during pregnancy avoiding a well-known issue reported for somatic cell nuclear transfer and other techniques [40, 41]. In addition, the production of mutant animals was remarkably high. We found that 50% of *in vitro* produced blastocysts showed some type of mutations. This result was confirmed after birth, since from 22 born lambs analyzed by T7EI assay and sequencing, ten were mutant (45.5%). Five of them (i.e. 50% of mutated ones) were biallelic KO inducing a myostatin deficient phenotype. The performance showed in this study with CRISPR/Cas9 system is clearly higher than the reported for other previous techniques in farm animals. In general, very low outcomes of pronuclear microinjection, several difficulties with nuclear transfer, and the technical challenge to produce lentiviral vectors of good quality, have limited the spread of transgenic technologies to new laboratories around the world [42]. Previous work has shown that mice or rats mutated in the *mdx* gene generated using CRISPR/Cas9 [43] or TALENs [44] respectively, showed mosaicism that resulted in some cases in absence of transmission of the mutation to the offspring. For our mutated lambs, their age and time of gestation after mating preclude this analysis. Nevertheless, the fact that both skin and muscle, developing from ectoderm and mesoderm, showed mutations and that the percentage of DNA mutated analyzed using the T7EI assay showed a high degree of mutation, it is likely that the mutations will be transmitted to the offspring by at least some of the founders. Our results show that CRISPR/Cas9 is an easier, faster and more effective technique to produce a high proportion of mutant animals, including biallelic founders, when compared to somatic cell nuclear transfer, the only technique available up to date in sheep to perform targeted gene KO.

Off target effects using CRISPR/Cas9 system as well as ZFNs or TALENs could be a problem when trying to obtain KO animals for a specific gene. However it should be pointed out that most of these off effects have been shown in cells transfected *in vitro* [45] and not in genetically-modified animals [15, 46], possibly due to a larger amount of transfected nucleic acids as compared to microinjection. Furthermore, breeding of mutated founders with WT partners results in dilution of the potential off target mutations in the offspring. In our case, we only found one non coding region that was mutated in two animals. Thus, the potential off effects seem low in this report. If more specificity is desired, strategies have been designed to overcome this difficulty, such as the generation of Cas9 mutants able to cleave only one DNA strand [47] or shorter sgRNAs [48] that show lower off effects.

Our study describes the use of CRISPR/Cas9 gene editing system in sheep, targeting the *MSTN* gene to modify the phenotype related to body weight and growth rate. Myostatin mutant lambs produced in this study were significantly heavier than control lambs, and moreover mutant animals developed normally until today. Myostatin have been previously modified by genetic engineering in ruminant species using technologies such as ZFN and TALEN [49, 50]. However, the use of the CRISPR/Cas9 system is easier and is now available for livestock production. Interestingly, while no differences were found at birth in body size and weight among homozygous KO lambs and wild type counterparts, KO lambs were 20–30% heavier 60 days later with no differences in body size. The production of newborns with low body weight at birth and faster growth rate, obtaining heavy lambs showing similar body size, represent an interesting productive feature in terms of lambing ease, meat production, and dressing percentage for livestock industry. Moreover, double muscle animals had shown a lower content of intramuscular fat, with more unsaturated fatty acid [51]. These characteristics make this meat healthier for the consumers, reaffirming the relevance of applying this technology to improve these kind of traits [52]. In addition, this mutation applied in Merino superfine animals could exemplify a new system for genetic improvement programs as never described before, with specifically designed dual or multi-purpose animals to produce more meat maintaining very high quality wool production. It is interesting to point out that the use of gene editing technologies, with the generation of mutations without introducing exogenous DNA, overcome the issue of introducing an exogenous gene into the animal genome. In this sense, a spontaneous mutation of *MSTN* gene that modify myostatin expression is present in some cattle breeds (e.g. Belgian Blue and Piedmont breeds) [53], as well as in sheep (e.g. Texel breed) [54] and goats [55]. Since these animals are already present in livestock production, their meat has been consumed for a long time. The easy access to CRISPR/Cas9 technology by more laboratories, together with more reasonable regulatory standards for its approval since no exogenous genes are inserted, suggests a promising future for gene editing systems in animal industry.

The CRISPR/Cas9 technology will also be likely used in the future to generate lambs harboring exogenous sequences to produce recombinant proteins with much higher efficiency through the introduction of these sequences by homologous recombination into *loci* that are permissive for frequent and high transgene expression, such as it has been done in other species using the *Rosa26* locus [35].

In conclusion, our study reports the production of healthy myostatin KO lambs using the CRISPR/Cas9 system in an efficient way to increase muscle growth and body weight. CRISPR/Cas9 system could be easily applied to obtain mutant farm animals for several genes of productive or biomedical interest.

## Supporting Information

S1 Table Comparison of skin and muscle DNA sequencing. (DOC)

S2 Table Primers designed for the amplification of the potential off target sites. Seven loci were defined, amplified and sequenced.
(DOC)

S1 Fig Myostatin western blot. Muscle biopsies from 2 representative animals, one heterozygous (heteroz.) for a frameshift mutation and a WT copy in the second allele and another WT for both alleles (#51 and 54, respectively, the numbers of animals correspond to those of Fig 3) analyzed by western blot using an anti- myostatin monoclonal antibody. After stripping of the anti-myostatin antibody from the membrane, an anti-GAPDH was used as loading control. One representative western blot experiment out of three performed with the same results.
(TIF)

S2 Fig Development on Day 105 of gestation in fetusses derived from CRISPPR/Cas9 inyection into zigotes. No statistical differences were found among the three genotypes for any of the variables.
(TIF)

S3 Fig Morphometric variables in wild-type, homozygous KO and heterozygous KO lambs produced by CRISPR/Cas9. (TIF)
