# Supplementary material for: Efficient Generation of Myostatin Knock-Out Sheep Using CRISPR/Cas9 Technology and Microinjection into Zygotes
Source: PLoS One. 2015 Aug 25;10(8):e0136690. doi: 10.1371/journal.pone.0136690 (PMC4549068; doi:10.1371/journal.pone.0136690)
Supplement: S1 Table — (DOC) [file pone.0136690.s001.doc]

**S1 Table. Comparison of skin and muscle DNA sequencing.**

|  | skin  PCR amplicon | muscle  PCR amplicon | muscle  TA cloning |
| --- | --- | --- | --- |
| #40 | ins 14bp | ins 14bp  +  ins 1bp | ins 14bp x2  +  ins 1bp x 1 |
| #47 | del 2 bp  +  del 8bp | del 2bp  +  del 8bp | del 2bp x6  +  del 8bp x 4 |
| #49 | del 5 bp  +  del 6bp | ND | del 5bp x5  +  del 6bp x3 |
| #50 | ins 2 bp  +  del 6bp | ins 2bp  +  del 6bp | ins 2bp x5  +  del 6bp x6 |
| #56 | del 4bp  +  del 6bp | del 4bp  +  del 6bp | del 4bp x4  +  del 6bp x6 |
| #57 | mosaic | ND | ins 25bp x2  +  del 14bp + ins 6bp x10 |
| #60 | mosaic | del 4bp  +  del 16bp | del 9bp + ins 5bp x5  +  del 27bp + ins 11bp x3 |

DNA from skin or muscle biopsies was genotyped by performing sequencing of *MSTN* PCRs (PCR amplicon). For each allele the genotype is depicted in the upper and lower lines of each case. Del=deletions. Ins=insertions. Mosaic means several complex mutations that could not be precisely determined. For muscle DNA, genotyping was also performed by TA cloning of *MSTN* PCR products, transformation of bacteria and sequencing of isolated bacterial colonies. X followed by a number depicts the number of colonies in which the different genotypes were observed.

Note that there is correlation between the mutations observed in skin and muscle using PCR amplicons and between the PCR amplicon and TA cloning sequencing for muscle. The only exception is for animal #40 for which there is an additional mutation observed using TA cloning (ins 1bp). Animal #60 may seem to show a discrepancy between PCR amplicon and TA sequencing but this is not the case and it is only due to the fact that the deletions defined using PCR amplicon sequencing are the net result of deletions and insertions defined by TA cloning (i.e. a deletion of 4bp by PCR cloning is the result of a deletion of 9bp and the insertion of 5bp and for the other allele that shows a deletion of 16bp this is the result of a deletion of 27bp and an insertion of 11bp).
