# Supplementary material for: Efficient Generation of Myostatin Knock-Out Sheep Using CRISPR/Cas9 Technology and Microinjection into Zygotes
Source: PLoS One. 2015 Aug 25;10(8):e0136690. doi: 10.1371/journal.pone.0136690 (PMC4549068; doi:10.1371/journal.pone.0136690)
Supplement: S2 Table — Seven loci were defined, amplified and sequenced. (DOC) [file pone.0136690.s002.doc]

**S2 Table. Primers designed for the amplification of the potential off target sites.** Seven loci were defined, amplified and sequenced.

| Off target region number | Forward Primer 5’- 3’ | Reverse Primer 5’ - 3’ |
| --- | --- | --- |
| 1 | ACCAGGAATAAAGTGGCGGG | GCGAGACCCCAAAGTGAGAA |
| 2 | TAAACCCACCCGTGGAATCG | TTGGCGGTGGCATTCACTAT |
| 3 | TGATACAGGGGGAGCACACT | CCTCTGGCATCCTCACACAT |
| 4 | AGTACAAAGTGCAGGGCAGT | GGCTTCAACTCAGGGCACTA |
| 5 | GTTCTGACTGGTCTCGGGTG | GCATATTCTGTGACCCGCCT |
| 6 | AGGAATCATGGAACAATGGACTG | TTTTGGATGGACATGGGCGT |
| 7 | AGAATGACCTGCAAGCCCTC | GGCTGTGCCTTGACTGTTTG |
