# Supplementary figures and images for: Efficient Generation of Myostatin Knock-Out Sheep Using CRISPR/Cas9 Technology and Microinjection into Zygotes
Source: PLoS One. 2015 Aug 25;10(8):e0136690. doi: 10.1371/journal.pone.0136690 (PMC4549068; doi:10.1371/journal.pone.0136690)

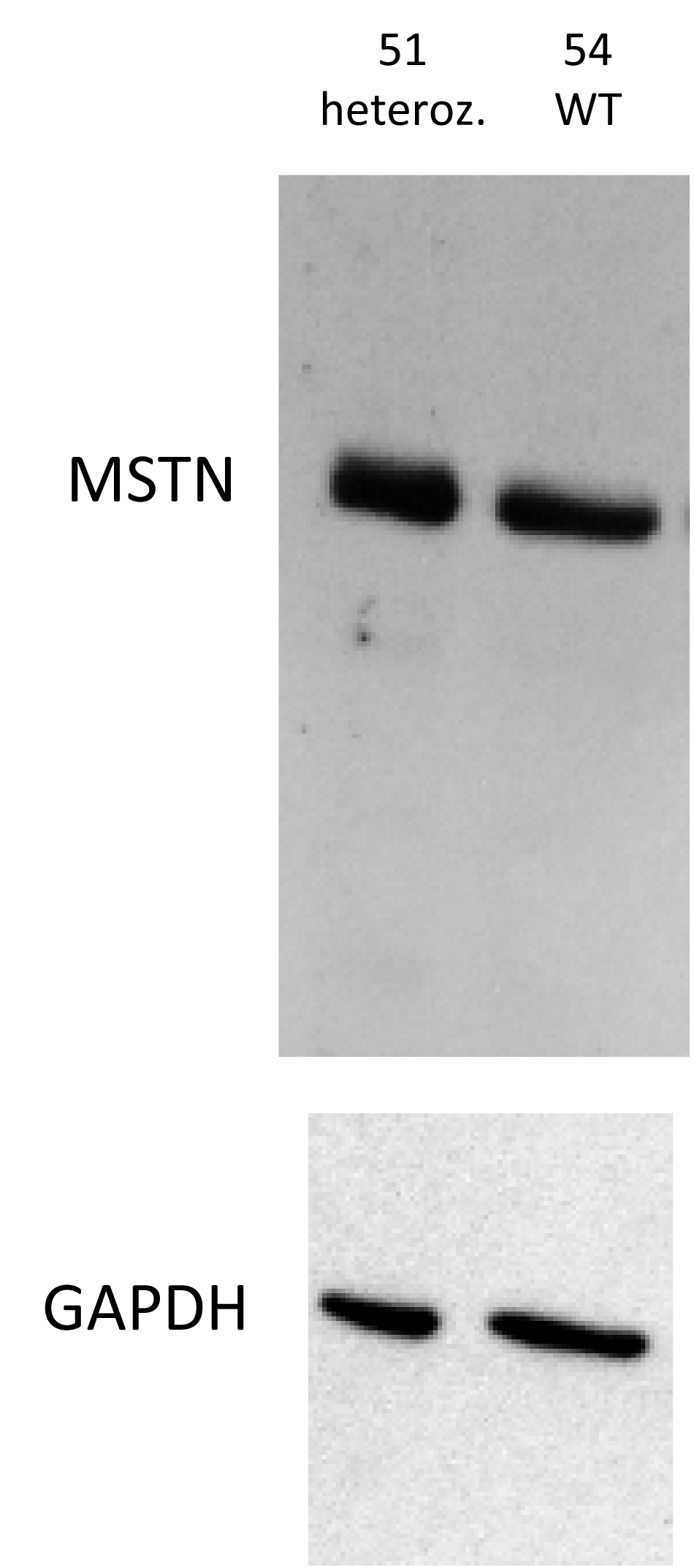

Supplement: S1 Fig — Muscle biopsies from 2 representative animals, one heterozygous (heteroz.) for a frameshift mutation and a WT copy in the second allele and another WT for both alleles (#51 and 54, respectively, the numbers of animals correspond to those of Fig 3) analyzed by western blot using an anti- myostatin monoclonal antibody. After stripping of the anti-myostatin antibody from the membrane, an anti-GAPDH was used as loading control. One representative western blot experiment out of three performed with the same results. (TIF) [file pone.0136690.s003.tif]

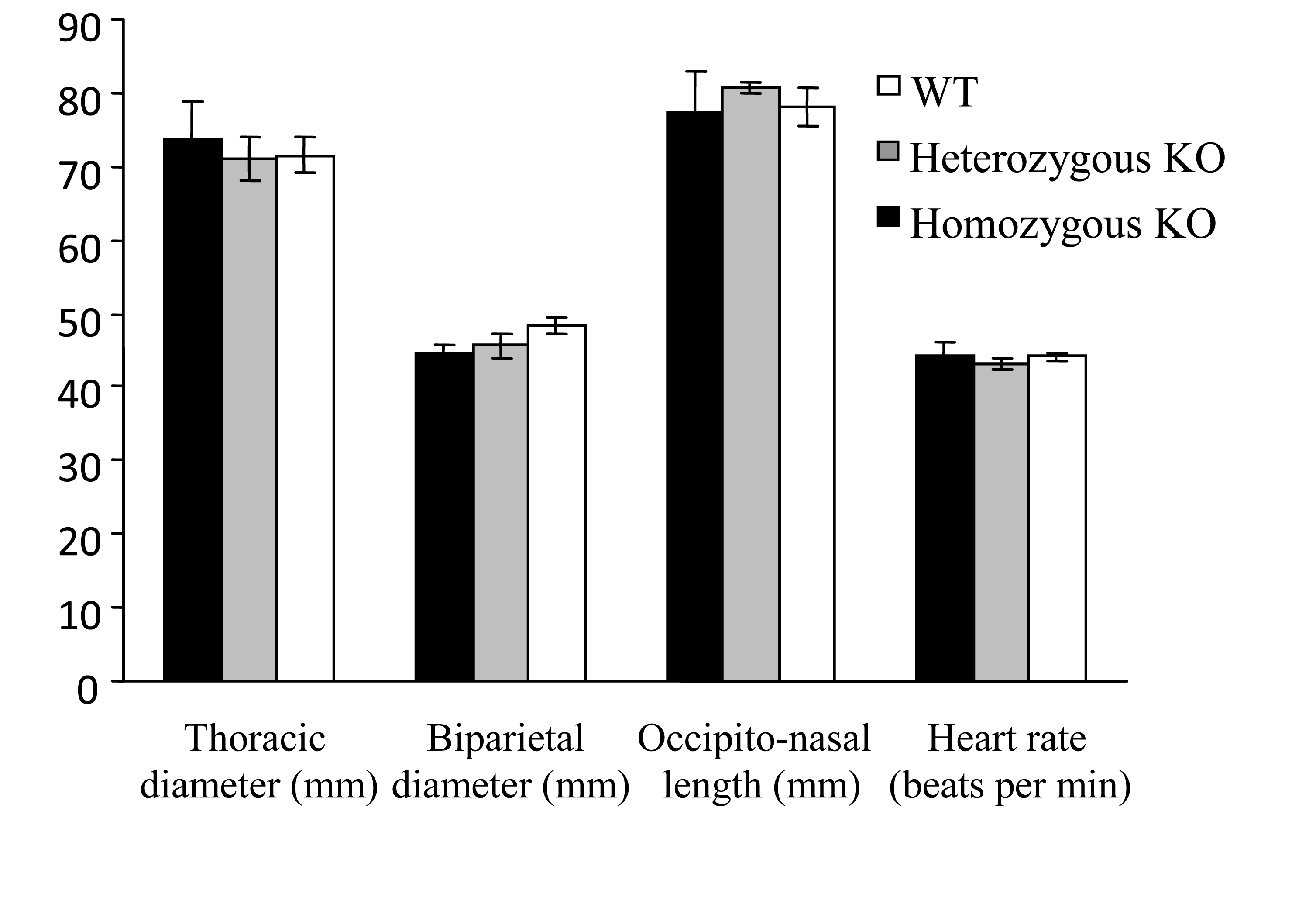

Supplement: S2 Fig — No statistical differences were found among the three genotypes for any of the variables. (TIF) [file pone.0136690.s004.tif]
